# Co-lethality studied as an asset against viral drug escape: the HIV protease case

**DOI:** 10.1186/1745-6150-5-40

**Published:** 2010-06-17

**Authors:** Sophie Brouillet, Thomas Valere, Emmanuelle Ollivier, Laurent Marsan, Anne Vanet

**Affiliations:** 1 Atelier de Bio Informatique, F-75005, Paris, France; 2 Université Paris Diderot, Paris 7, F-75013, Paris, France; 3 Université Pierre et Marie Curie, UMR 7138, Paris 6, F-75005, Paris, France; 4 Université de Versailles Saint Quentin, UMR 8144, Équipe AOC, Laboratoire PRiSM, F-78035, Versailles, France; 5 INSERM, U973, F-75013, Paris, France

## Abstract

**Background:**

Co-lethality, or synthetic lethality is the documented genetic situation where two, separately non-lethal mutations, become lethal when combined in one genome. Each mutation is called a "synthetic lethal" (SL) or a co-lethal. Like invariant positions, SL sets (SL linked couples) are choice targets for drug design against fast-escaping RNA viruses: mutational viral escape by loss of affinity to the drug may induce (synthetic) lethality.

**Results:**

From an amino acid sequence alignment of the HIV protease, we detected the potential SL couples, potential SL sets, and invariant positions. From the 3D structure of the same protein we focused on the ones that were close to each other and accessible on the protein surface, to possibly bind putative drugs. We aligned 24,155 HIV protease amino acid sequences and identified 290 potential SL couples and 25 invariant positions. After applying the distance and accessibility filter, three candidate drug design targets of respectively 7 (under the flap), 4 (in the cantilever) and 5 (in the fulcrum) amino acid positions were found.

**Conclusions:**

These three replication-critical targets, located outside of the active site, are key to our anti-escape strategy. Indeed, biological evidence shows that 2/3 of those target positions perform essential biological functions. Their mutational variations to escape antiviral medication could be lethal, thus limiting the apparition of drug-resistant strains.

**Reviewers:**

This article was reviewed by Arcady Mushegian, Shamil Sunyaev and Claus Wilke.

## Background

RNA viruses alone include 350 different human pathogens. Most are the agents of newly emerging diseases. Recent concerns for actual or feared pandemics (SARS, avian flu, or swine flu viruses) all raised the challenge to quickly come up with solutions. Worldwide, over 100 million influenza cases occur each year, 170 million people carry HCV, and 33 million HIV. RNA viruses generally have very high mutation rates as they use polymerases which cannot find and fix mistakes, and are therefore unable to conduct genomic repair of damaged genetic material. Under selective pressure, this error-prone replication can confer drug resistance. Since AIDS appeared, many new drugs have been created and used against RNA viruses, which in turn readily evolved drug-resistant strains, a now predictable process and an unprecedented public health issue. HIV mutant strains that escape antiviral compounds have been extensively documented [1], and one of influenza's main treatments, tamiflu, was quickly escaped from by an influenza strain which then spread surprisingly fast across the planet [2]. It now becomes clearer that future antiviral strategies should address this issue from the outset, aggressively striving to prevent viral escape. To deal with this, several directions have been explored since a decade: the structures of resistant proteins [3], second generation drugs that can bind resistant proteins [4], drug target polymorphism analysis in order to define "super drugs" [5], definition of new targets, such as protein backbones [6] or dimeric proteins' monomers, in order to block them before dimerisation [7]. Overall, although individual case solutions were found, no general solution has emerged yet.

It is now documented that drug resistance is due to at least one mutated amino acid, so researchers have recommended, that invariant viral amino acids should be targeted by future new drugs [8]. The rationale is that mutations in invariant positions always damage a critical biological function, resulting in non-replicative viruses. For exemple, zanamivir is a broad inhibitor of neuraminidase, an influenza glycoprotein, probably because it interacts with invariant amino acids inside the enzyme active site [9]. Ceccherini *et al*. [10-12] identified new conserved residues for the HIV protease, reverse transcriptase and integrase, and mapped them on the 3D protein structure to help to design a new structure-based escape-resistant inhibitor.

Unfortunately, sometimes, invariant positions are too few to constitute a proper docking site for a drug. For example, 63% of the HIV protease amino acids are variable [13]. In an effort to gather enough replicativity-critical amino acids for a possible docking site, we propose to also target synthetic lethals (SL, or co-lethals). Synthetic lethality is the lethality resulting when two, individually survivable, mutations, are co-present [14]. Each mutation is then called a synthetic lethal. Analysis of SLs is a powerful tool to understand genetic interactions and essential metabolic pathways. Synthetic lethality has been extensively used to study gene products' interactions in the secretion pathway in yeast [15], in bacteria [16] and even to identify anticancer agents [17]. It was noticed that, in many cases, the double mutants identified had their co-lethal mutations in 2 different genes. A few yeast studies analyzed protein structure-function aspects, revealing intragenic synthetic lethals [18], i.e. cases where the 2 co-lethal mutations occur in one same gene. Little SL research has been undertaken on viruses. Researchers were rather on a quest for the opposite situation, where one crippling mutation is rescued by a second, intragenic, suppressor mutation, the second restoring the function lost due to the first mutation [19,20]. They also found few, under-represented intragenic suppressors, which actually turned out to be SLs.

In line with our patented model [21] we chose to focus on intragenic synthetic lethals in the HIV protease. As mentioned above, the number of replicativity-critical amino acids available for drug docking should be maximized, invariant amino acids being not numerous enough, and one single SL couple being possibly too little. So we propose to add larger "SL sets" rather than one single SL couple. By "SL set", we mean a set of SL couples that are connected to each other. We choose to add also the invariant position being in the vicinity of the SL set. Indeed, a set of positions containing invariant amino acids plus an SL set may be large enough a target for putative antiviral drugs.

To test this anti-escape approach in practice, we study the HIV-1 protease from the B sub-type. This protein is a 99 amino acids homodimeric aspartic protease and its substrate-binding pocket includes the D25-T26-G27 catalytic triad and flap regions, which presumably open and close to allow entry and binding of substrates or inhibitors [22,23]. This protein has more than 60% of variant positions. We first aligned 24,155 amino acid sequences of the HIV protease. From this alignment, we determined all the couples of positions that were statistically never found mutated together, that we called SL and focused on the SL set, which are accessible to the solvent, and not too far apart spatially. We report here that our method has yielded 3 targets of respectively 4, 5 and 7 amino acids. Unlike currently documented drug targets, which mutate and escape drug treatments, these targets should be conserved, otherwise viral replicativity would suffer, impairing viral pathogenicity. More generally, this process can of course be used against other HIV proteins, other RNA viruses, or any highly variable agents.

## Results

### Graph of the accessible and spatially close synthetic lethal clusters

The first step is to define which couples of protease positions are synthetic lethal. To do so, we analyzed, from patients, 24,155 protease amino acid sequences of HIV-1 subtype B. Some patients had been previously treated with antiviral drugs, and some never, so the pool of sequences more appropriately represents the actual viral diversity. We kept all the sequences in one same set for four different reasons. Firstly, an untreated patient can get a viral sequence - possibly highly mutated - from a treated patient. Secondly, treatment is a selection pressure but other pressures can select other mutations appearing in both treated and untreated sets. Thirdly, our dataset can be enriched with plenty of mutations from unknown patients with unknown treatment histories. Finally, we wanted to create a tool that is robust enough to be used on other RNA virus databases that are less documented than HIV. When sequences occurred in multiple copies in the sequences collection, we kept all these redundant copies, assuming redundancy may reflect biological fitness (mostly identical copies from different patients, rather than multiple samples from the same patient). We did not use clonal data because it is less informative: not enough sequences, and not mutated enough. These 24,155 sequences were aligned. From this alignment, we found 25 positions that display changes in less than 0,3% of the sequences. We called those positions "invariants". Ceccherini-Silberstein *et al*. [10], who defined invariant positions as displaying changes in less than 1% of the sequences, found 46 of those positions. All our 25 invariant positions were totally included in the Ceccherini-Silberstein invariant set of protease inhibitor (PI) treated patients. Stanford university HIV drug resistance database [13] described 36 positions represented in less than 0.5% of the HIV subtype B sequences from PI treated patients. Our 25 invariant positions are also all included in this group. The invariants positions were then set aside momentarily; and we compared the 74 variant positions in pairs (2,701 couples).

To detect the SL couples in these 74, we first examined the distribution of the p-values of the χ^2 ^test associated to each of these couples. Using a 0.5% False Discovery Rate (FDR) threshold, we identified 290 synthetic lethal couples (10.7%). These 290 couples span 70 protease positions out of the 74. The 4 remaining positions are not represented in these couples. We named "total SLs" the graph made of 70 nodes representing the 70 protease positions, and 290 edges representing the 290 relations "is co-lethal with" linking the synthetic lethal positions (data not shown).

Three other teams have used different methods (information theory [20], chi^2 ^studies [19] Bayesian networks [24]) to identify couples of HIV protease co-evolved positions. Amongst other results, they all found few synthetic lethal couples, around 15. Although unlike other teams, we did not discriminate the proteases from treated versus non-treated patients when collecting our initial lot of sequences, all these SL couples are included in our 290 SL couples and none of them are included in our SL sets. Our method therefore enables us to find the 15 couples already described by other teams using other methods.

**Figure 1:**
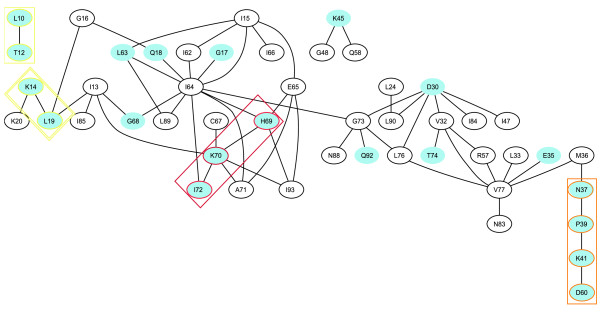
**Spatially close SLs**. The whole graph represents the "spatially close SLs". The blue-shaded ovals, represents only the "accessible, spatially close SLs". Each oval contains the amino acid (one-letter codename) found in the HIV-1 protease ancestral sequence, followed by its position in this sequence. The accessible residues are all shaded in blue. The black edges linking the nodes mean "is co-lethal with and is within 10 Angström from". The four orange, boxed amino acids (N37, P39, K41, D60) are the locked targets jointly called SG flap. The three red, boxed amino acids (H69, K70, I72) are the locked targets called SG canti. The two yellow, boxed amino acids (L10, T12) are the locked targets called SG fulc1 and the two yellow double-boxed amino acids (K14, L19) are the locked targets called SG fulc2. SG fulc1 plus SG fulc2 are called all together SG fulc. The graph was built with Graphviz software.

The distances between the two positions of each couple were calculated using the 3D protease structure PDB ID:1HSG[25]. The resulting distance graph was then superimposed to the "total SLs" graph. To be accessible by a putative drug, amino acids should fulfill two preconditions: to be spatially close enough to possibly form a pocket structure, and to be solvent-accessible (on the outside of the protein). Thus we chose to retain only the clusters of close positions, by only keeping the edges that indicate less than 10 Angström of distance between two nodes. This resulted is a drastically reduced graph, of "spatially close SLs", shown Figure 1, going from 290 nodes to 48.

From the "spatially close SLs" graph, we selected the solvent accessible positions. (shown Figure 1 as the 20 shaded ovals). Visibly, only 4 clusters contained interlinked exposed positions, boxed in Figure 1, encompassing 11 nodes (shown as colored, orange red and yellow ovals) and 7 edges (black continuous lines). These are 4 separate, intra-connected subgraphs (or SG). These 4 SL sets are referred to as SG-flap (4 nodes, shown as orange ovals), SG-canti (3 nodes, red), SG-fulc1 (2 nodes, yellow) and SG-fulc2 (2 nodes, yellow). The combination of SG-fulc1 plus SG-fulc2 is named SG-fulc. The names were given retrospectively, as reminders of 3 specific regions of the protease 3D structure [26,27]. Namely, SG-flap includes 3 positions found in the protease flaps. SG-canti includes positions found in the protease cantilever. And all positions of SG-fulc are in the protease fulcrum. No subgraph is part of the catalytic site [28]. The 4 clusters we identified can now be complemented by their neighboring, accessible, invariants.

### Extending the SL sets: the flaps and cantilever

**Figure 2:**
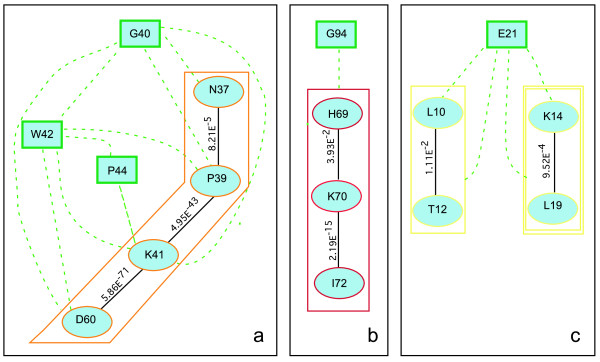
**locked targets (accessible, spatially close locked sets)**. All nodes shown here are accessible and spatially close amino acids The nodes in ovals are the accessible synthetic lethals which were shaded in blue on Figure 1, boxed with the same colour codes and linked by black edges. Each black edge means "is co-lethal with, and within 10 Angström from". The corresponding p-value is shown above each edge. Invariant amino acids are boxed in green, linked by dotted green edges meaning "is within 10 Angström from". All the nodes are shaded in blue because they are all accessible. Each invariant is also linked to all the other nodes by implicit edges, not shown, for the sake of clarity. The locked target represented subgraph "a" is called SGI flap (orange+green) The locked target represented subgraph "b" is called SGI canti (red+green) The locked target represented subgraph "c" is called SGI fulc (yellow+green) The graph was built using the Graphviz software.

For an anti-protease drug to be active, its docking must block a vital protease function. To compromise viral escape, it should dock on an invariant position or on an SL set. As described in Materials and Methods, we kept the invariant positions aside, as obviously lethal, when searching for synthetic lethals. But invariant positions are of course prime target candidates for the docking of an antiviral agent with limited escape possibilities. So we added all the invariant positions (shown as green rectangles shaded in blue on Figure 2) that are accessible and close, to the 4 locked sets (shown as ovals on Figure 2).

By the addition of invariants, we obtained 4 locked sets. But how much less escape-prone, how much more "locked? To quantify this, and rank graphs in terms of how closer to a maximal clique they were brought, we defined a connectivity coefficient, C, in Materials and Methods. C varies between 0, for a graph with no edges, and 1, for a graph where all the possible edges between nodes are present, i.e. a maximal clique. The closer C is to 1, the least escape-prone the group is.

**Figure 3:**
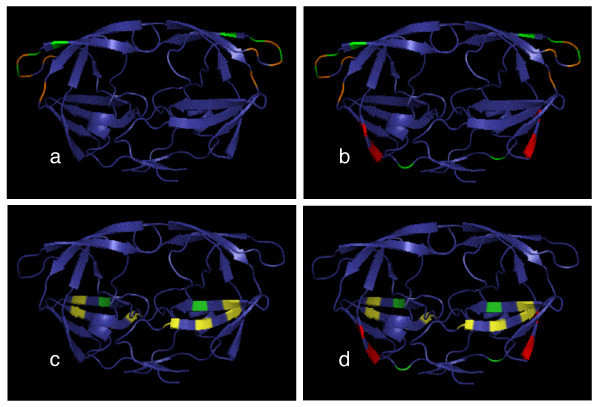
**3D view of the locked targets**. 3D representation of the HIV-1 homodimer (1HSG) protease. Same colour codes as Figures 1 and 2. a: SGI flap (4 orange SLs + 3 green invariants) b: SGI flap + SGI canti (2 red SLs + 1 green invariant) c: SGI fulc (2 yellow SLs + 1 green invariant) d: SGI fulc + SGI canti (4 yellow SLs + 1 green invariant) The 3D molecules were built by pymol software.

The full Figure 2 graph has 16 nodes and 72 edges (7 in black plus 65 implicit edges) linking each invariant position to all the others. For the sake of clarity only the 15 implicit links within a 10 Angström distance are represented (as dotted green lines). SG-flap is now included in a larger connected subgraph named SGI-flap (7 nodes/18 edges including 3 in black, 10 in dotted lines, and 5 implicit edges Figure 2.a). The connectivity coefficient is increased from C_SG-flap _= 0.5 to C'_SGI-flap _= 0.71. SGI-flap has 3 invariant amino acids, out of which 2 (G40, W42) are within 10 Angström of each other, G40 is within 10 Angström of the whole SG-flap residues and W42 is within 10 Angström of (P39, K41, D60) from SG-flap residues. The whole SGI-flap set is an interesting result since its amino acids (N37, P39, G40, K41, W42, P44, D60) are all accessible, can't all freely mutate without damaging replicativity, and are close enough in space to imagine designing a single drug to target several of them. Figure 3a shows these amino acids on the dimerized 3D structure, using the same color codes as Figure 1 and 2: four orange SLs and three green invariants. It is important to note here that each of these 7 positions does bear indispensable viral functions, namely:

- Mutation in position 40 provokes a total loss of the protease activity [29].

- Mutation in either position 39 or 60 provokes a 50% drop in the protease activity [29]

- Out of the 105 variants analyzed by Martinez et al. [30], those with a mutation in position 37 display in average a 50% drop in the protease activity vs. wild type. Double mutants in positions 37 and 39 show a 75% drop vs. wild type, consistent with the possibility positions 37 and 39 might be SLs.

- Position 42 seems correlated to the flaps opening, and positions 39, 40 and 44 are very flexible, and positively required for the flaps to open [31].

- Double mutants on K70E and R41T replicate poorly [32]; an important piece of information for us since we report these 2 positions are SL (cf next paragraph).

### Extending the SL sets: the cantilever and fulcrum

A look at SG-fulc yields complementary target recommendations. On SG-fulc, amino acid L10 is interesting because it is linked to the 3 other SLs (T12, K14, L19) as well as to invariant E21. The latter is close to all SG-fulc positions. T12 happens to be less than 10 Angström (not shown) from the other 4. Furthermore, if distance is not taken in account, the amino acids (L10, K14, L19) are a maximal clique, so at most one may be found mutated. Overall, we identify a cluster of 5 amino acids with a connectivity coefficient of C'_SGI-fulc _= 0.8, which are all close and amongst which at most two may freely mutate. This set, (L10, T12, K14, L19, E21), called SGI-fulc, is therefore a good target for a putative drug (Figure 3c). It is interesting to note that position 10 can have a secondary mutation conferring resistance [33] and that mutations in position 12 occur more often in treated patients than in drug-naive patients [34].

### Are the locked targets we described actually druggable pockets?

We studied this using Q-siteFinder, a software program from the University of Leeds [35]. Q-siteFinder takes a 3D structure input and outputs its top 10 ligand-binding sites. Two out of the 10 ligand-binding sites in the protease overlap 2 of our locked targets (SGI-flap and SGI-fulc). Five out of seven positions in SGI-flap (39, 40, 41, 42, 60) are in the pocket of one of the 10 sites defined by Q-siteFinder (39, 40, 41, 42, 43, 58, 59, 60, 61, 62). Three out of five positions in SGI-fulc (12, 14, 19) are in the pocket of one of the 10 sites defined by Q-siteFinder (12, 13, 14, 19, 65, 66, 67, 68).

## Discussion

All the amino acids in the first locked target (subgraph SGI-flap on Figure 3a) are part of the protease flaps' external loop. The flaps being mobile structures [36] that open out to let the substrate pass, one can imagine that a molecule drug-designed to bind the external loop of the protease flaps could block their mobility, therefore keeping the protease from processing its substrate. This idea was also developed by Perryman *et al*. using molecular dynamics [37]: they also proposed to affect the flaps' mobility. Our approach is in agreement with theirs, and we believe this group to be a very good candidate for future drug targeting.

The method described in this manuscript enables us to define which amino-acids would be the most adequate drug targets to limit or avoid escape. But this method does not tell us whether these amino-acids define an actually druggable pocket. To check that, we used the Q-siteFinder software [35], which takes the 3D structure of a protein as input, and outputs its ligand-binding sites. Out of the 3 SL locked targets we describe, 2 (SGI-flap and SGI-fulc) closely overlap two sites revealed by Q-siteFinder for the HIV protease, on respectively 5/7 and 3/5 positions. This result suggests our SL locked targets SGI-flap and SGI-fulc actually are druggable pockets.

Since all our results use the "ancestral sequence" as a starting point, a relevant methodology control would be to check whether our above results stand if another acceptable starting point sequence is used. So we used the same method with the historical reference in medical studies, the HIVLAICG sequence [38] (see methods), instead of the "ancestral sequence". The results are a smaller graph (not shown), equal to Figure 2 subgraphs SG-canti and SG-fulc plus 2 amino acids, (E34, E35). We believe both approaches are complementary and support each other, as mutual controls, since our recommendation to target SG-canti + SG-fulc is conserved to a great extent.

From a general standpoint, it seems obvious that the different branches of one same phylogenetic tree can have their specific, own synthetic lethals. These synthetic lethals should not be mixed, since they're only usable targets in their relevant phylogenetic branch. In the case of HIV-1, they are three main branches are groups M, N and O. The branch of sub-group M is further divided in 7 sub-groups, including sub-group B. We choose to work on sub-group B because it has the more published sequences (over 20,000 for the protease) and mutations. Although there is no simple algorithm today to build a tree from tens of thousands of sequences, it would be interesting to study the phylogenic tree of our pool of sequences in parallel with our synthetic lethality study. However from an evolutionary point of view, we believe that the positive co-evolution of two sites (both positions always mutate together) is not the same issue as exclusive co-evolution (both positions can mutate but never together) Indeed, when examining the propagation of 2 mutated positions in a tree, two situations can be distinguished: either they are co-present in the majority of the sub-trees, which tends to indicate noise (since there is no segregation); or they are co-present only in the leaves of one part of the tree, which tends to indicate true co-evolution. Inversely, when studying a case of exclusive co-evolution, only one of these two situations is possible. Indeed, by definition of synthetic lethals, both mutations cannot be part of the same sub-tree since their co-presence is lethal. So the tree resulting from this situation is impossible to analyze, since one should analyze all the sub-trees in order to find the missing branches - which corresponds to the statistical study we conducted here.

Our method treats variation in all positions as bi-allelic (mutated versus non-mutated genotype). Arguably, it would seem more informative to take into account the actual amino-acid changes. This would entail choosing a score matrix - most score matrixes do take into account the physico-chemical specificity of each amino-acid so as to decide whether the mutated one is similar or not to the initial one. But the mutations involved in the resistance to anti-protease drugs do not follow the same rules. Therefore we do not think using an existing score matrix of the Blosum or Pam type would be more informative.

Finally, the fact a synthetic lethal co-mutation is never observed statistically does not mean such a co-mutation is completely impossible, hence our use of the expression "potential SLs" in cases with no experimental replication data. Our results indeed include cases where synthetic lethals were actually mutated (although this occurred in less 0.02%- 0.5% of the sequences, data not shown). So if these sequences truly represent replicative viruses, as opposed to unfiltered artifacts, then there is a risk that blocking some synthetic lethal couple with a new drug could create a selection bottleneck [39,40], forcing viral evolution towards more resistant strains. If such new escape mutants were to appear, they should carefully be monitored by sequencing at least their synthetic lethal positions, as a first step in further refining the antiviral strategy.

In any case, our method has the advantage to indicate the comprehensive list of all possible protease locked targets, the best targets to minimize or eliminate viral escape. Even if locked-set-designed anti-viral drugs were to somehow elicit escape mutants, the other (non-locked) amino acids would remain wrong targets, since viral escape seems nearly guaranteed with them: the corresponding multi-mutant HIVs are already in nature and their sequences in worldwide databanks.

## Conclusions

The constant rise of drug resistant RNA viruses is a reason to start looking for therapeutic strategies that minimize or eliminate viral escape. We described here a method to identify targets that may be involved in essential viral functions. These are what we call locked targets: spatially close, accessible viral invariant positions and/or potential synthetic lethals (SLs are groups of survivable mutations which are lethal whenever any two co-occur). Due to these two features, vital function and invariance, these targets are unique in that they might minimize or prevent viral escape. Our application to the HIV protease yielded 3 locked targets that are accessible, compact enough to possibly dock a drug, and are all outside of the enzyme's active site; whereas to date, all 9 existing anti-protease drugs were competitive inhibitors that bind the active site. The first locked target we closed in on is made of 7 amino acids positioned between the protease flaps and cantilever. The second locked target we detected is made of 5 amino acids in the protease fulcrum. The third locked target is composed of 4 amino acids located in the protease cantilever region. These three locked targets altogether contain 16 protease amino acid positions. Biological evidence regarding 10 positions out of these 16, provided in the Results section supports that those 3 locked targets are strategic drug targets. This because it seems a virus cannot easily mutate these targets to escape, as our statistics significantly exclude free co-mutations within those targets. While our method made no a priori assumptions on the positions or sequence sets, it revealed locked targets that bear essential biologic functions, which validates the starting hypothesis linking SLs to essential biological functions.

We believe this method can be used against any variable protein, for identification of the best locked targets.

Obviously, the approach described here can be used for the other HIV-1 [41] and HIV-2 genes, but also for other viruses such as HCV [42], SARS-coV or influenza [43], or indeed for any quickly variable protein sequence. The large sequence banks needed for the statistics exist already, or can expand quickly due to the speed improvements in mass sequencing technology.

## Methods

### Alignment of the sequences

The pol gene codes for three proteins: the protease, the reverse transcriptase (RT) and the integrase, containing respectively 99, 560 and 288 amino acids.

We pooled all of the amino acid sequences, whole or partial, of subtype B HIV-1 Pol from three databases: SwissProt, Los Alamos National Library [44] and Stanford University HIV Drug Resistance Database [13]. We thus collected over 70,000 Pol sequences from patients that are treated, non treated, or of unknown treatment history.

Using a reference amino acid alignment of 438 HIV-1 protease downloaded from the Los Alamos National Library [45], we built a profile with HMMER [46] package using hmmbuild and hmmcalibrate function.

Out of these 70,000 sequences, we first only kept those having at least 4 out of the 6 C-terminal protease motif (PQVTLW), or at least 4 out of the 6 N-terminal protease motif (GCTLNF), or both. This new set of over 20,000 sequences was then aligned on the abovementioned profile. The resulting alignment was used in order to create a new accurate profile for the alignment of small sequences. We then collected, from the initial 70,000 set, the partial sequences of 10 amino acids or more that had neither C-terminal nor N-terminal motif, but had at least 60% identity with the new profile. These partial sequences were then aligned on the new accurate profile. From this final alignment, we removed the 5 sequences that showed insertions.

We obtained one set of 24,155 protease sequences. Amongst the protease sequences, 11,206 sequences are represented only once, and the most frequent sequence was represented 257 times. 21,766 sequences are full length (contain the 99 positions of the protease).

### Choice of a reference sequence

In order to label positions as "mutated" and "wild type", we needed a reference sequence [47]. We first computed the consensus of our 24,155 protease sequences. But when we attempted to use it as our reference sequence, it misleadingly labeled as wild type some extremely frequent mutations. So we discarded that choice for a reference. The historical reference in medical studies HIVLAICG [GenBank:K02013] [38], was the first HIV-1 sequence ever published. HIVLAICG, though, is not the root of the phylogenic tree representing all the HIV-1 sequences. That root is the "ancestral sequence" [48], phylogenetically computed from subtype B sequences [45] and often the reference in biocomputing studies.

We observed that the sets of mutations defined vis-à-vis HIVLAICG or vis-à-vis the "ancestral sequence" were nearly identical (data not shown). Additionally, only two positions differ between these two sequences: positions 37 (N in the "ancestral sequence", versus S in HIVLAICG) and 41 (K versus R respectively). Overall, these arguments made us opt for the choice of the "ancestral sequence" as our reference. For control purposes, we also tried to use HIVLAICG as our reference in a separate experiment. It yielded very similar results.

### Identification of the potential synthetic lethal couples

Our algorithm proceeded as follows on the alignment obtained. For each of the 24,155 protein sequences, the first position was compared to the first position of the reference sequence, to determine whether it was mutated. Each position mutated vis-à-vis the reference was recoded as 0 and each conserved one as 1. The same process was iterated for the second position, and so on until the end of the sequence. Thus, each sequence was recoded into a series of 0 and 1. Then we got a "24,155 × 99" matrix which each box containing either a 0 or a 1.

To avoid mistaking "sequencing errors" for "mutations" in actually invariant amino acid positions, we set aside all the positions that display changes in less than 0.3% of the sequences. This cutoff of 0.3% was chosen here because it is the percentage of mutations we measured within three amino acids around the active site throughout our database. Using this threshold, we found 25 positions that display changes in less than 0.3% of the sequences. We refer to them as "invariants".

We focused on the 74 other positions, in a "24,155 × 74" matrix. We chose a χ^2 ^test to evaluate the mutual independence of positions in order to extract from this set the couples of potential synthetic lethality. We compared in pairs all these 74 positions (74 columns of the first matrix), noting the result in 2,701 (74*73/2) "2 × 2" matrixes. Each of these "2 × 2" matrixes (contingency table) contained four elements: the number of (mutated mutated) represented as (0 0) in the 2 columns that we are comparing in the first matrix, the number of the (non-mutated non-mutated) as (1 1), the number of the (mutated non-mutated) as (0 1) and the number of the (non-mutated mutated) as (1 0). In the null hypothesis that both positions are independent, using those 4 numbers, we performed 2,701 times, a one-degree-of-freedom χ^2 ^test, and the associated p-values were calculated for each of the couples of positions. Since we did multiple tests, in order to compare these p-value results and decide whether the results were significant or not, we studied the distribution of the p-values. Then using Benjamini-Hochberg's [49] and Storey [50] methods, we computed the false discovery rate (FDR) associated to the different p-values.

For each of the 100 alpha risk classes varying between 0 and 1 (each alpha risk class representing 1%), we computed the counts of the associated p-values and then built the associated histogram. Finally, the FDR associated to an alpha risk of 0.05 was computed with the following formula:(1)

with(2)

where n is the total number of p-values calculated, nb (α) the class count associated to a. Finally, we kept only the couples for which the p-value had an FDR lower than 0.05. The positions of these couples are therefore all statistically dependent in pairs.

Out of all these statistically dependent couples, in order to define synthetic lethals, we focused on the positions where the observed number of mutated/non mutated and non mutated/mutated couples was greater than the theoretically expected number if the two positions were mutating independently. This singled out couples of positions that statistically mutate less often together than what would be expected from a random behavior. This statistical dependency between positions is referred to below as the relation "seems synthetic lethal with", or, indifferently, "seems co-lethal with". The couples will be called SLs in the following, keeping in mind that formally they should be called "potential SLs" until biological data demonstrates actual co-lethality.

### Identification of the accessible positions

In order to define the accessibility of the amino acids to some putative external ligand - with a future drug in mind, using the 3D protease structure PDB ID:1HSG[25], we computed the surface accessible to the solvent, using the ASA software [51] available at RPBS [52], and considered "accessible" all amino acids with an accessibility threshold greater than 25%.

### Identification of locked sets

A set of elements (e.g. amino acids) linked by relations can be represented as a graph. The graph we designed for this study has one node for each protease amino acid position. The graph's edges bear the relation "is co-lethal with".

Calling N the number of nodes in a graph, the minimal number of edges is A_min _= 0 and the maximal number of edges is .

When a graph reaches the maximum number of edges, i.e. when all its nodes are interlinked two by two, it is called a maximal clique. This property is important when screening SLs because within a maximal clique, at most one amino acid may mutate freely, any second mutation being lethal.

To determine the maximal cliques contained in our graph, we used a program (Eric Coissac, personal communication).

Most SL sets are not maximal cliques, but some SL sets are quite close to being maximal cliques. Then we wanted to determine the degree of connectivity of a graph.

We defined C as  where A_obs _is the observed number of edges of the graph.

The closer C is to 1, the closer the graph is to a maximal clique, the set being so locked only one mutation is possible without lethality. Inversely, a value close to 0 indicates that all the positions considered may mutate without any lethality, the set is not locked, nor even an SL set, but just a plain set of amino acid positions.

Whenever a node is an invariant amino acid, it is by definition linked to all the other nodes. We indeed add invariants to SL sets in order to extend our targets into "locked sets". By "a locked set" we mean "an SL set plus all the invariant positions". Indeed, a set of positions containing invariant amino acids plus an SL set may be large enough a target for putative antiviral drugs. We call all these most conserved positions "locked" because they "can't escape" by freely mutating. Invariants are totally locked (no mutation is survivable), while SL sets are partially locked (some, but not all, mutation combinations are survivable).

When n invariant nodes are added to a graph of m nodes already linked by A _obs _edges, then the new connectivity coefficient C' is:(3)

The connectivity gained when invariants are added to an SL set is quantified by the increase from C to C'.

## Abbreviations

HIV: Human immunodeficiency virus; AIDS: acquired immunodeficiency syndrome; SL: Synthetic lethality; HVC: Hepatitis C virus; SARS: Severe Acute Respiratory Syndrome; SARS-coV: Severe Acute Respiratory Syndrome CoronaVirus; FDR: False Discovery Rate; PI: Protease Inhibitor; RT: reverse transcriptase; PDB: Protein Data Bank; SG: Sub-Graph; SGI: Sub-Graph + Invariant; Fulc: Fulcrum; Canti: Cantilever; 3D: Tridimensional

## Competing interests

The authors have a financial interest in patent application, which cover some of the work described in the paper.

## Authors' contributions

SB Carried out the sequence alignment, identified the synthetic lethal couples and the locked sets, performed the statistical analysis, wrote the method section and helped to draft the manuscript; TV helped with study design, provided comments and feedback on draft manuscript and translated it into English; EO created the database and was involved in drafting the manuscript; LM provided help in statistical and SL couples analysis; AV carried out conception, design and coordination, analysis and interpretation of the SL couples and locked sets, wrote the draft manuscript and gave the final approval of the version to be published. All authors read and approved the final manuscript.

## Reviewers' comments

### Reviewer's report 1

Reviewer 1: A.Mushegian, Stowers Institute, Kansas City, United States

### Reviewer's comment

The manuscript by Brouillet and co-authors presents a simple, direct and, in my opinion, promising strategy towards computational selection of druggable targets, based on the inference of intramolecular synthetic lethal pairs of amino acid substitutions. It is clearly written, and I do not have any major concerns with the technical side of the work. However, additional explanation of the following would be helpful.

The authors present their reasons for including all the data, except for incomplete and suspected-erroneous sequences. These reasons are compelling enough to me, and yet I am wondering whether the data are biased in some way. Does the assignment of synthetic lethals change if the dataset is made non-redundant? If we sample from the total dataset repeatedly?

### Authors' response

As a control, we indeed used a dataset of non-redundant protease sequences, to determine the SL sets and their deduced locked targets. The same three locked targets were found, but with fewer positions. The corresponding SGI-flap was constituted by the same SGI-flap positions except position 37. SGI-canti was also constituted by the same positions except position 69. And SGI-fulc lost 2 positions, 14 and 19. Since there was not much of a difference, we used the population more representative of biological fitness, as we explain in the manuscript.

### Reviewer's comment

A related issue is the application of the method to other molecules. The authors indicate that if there are not enough sequences (from another virus) in the databases, the new-generation sequencing methods may come to the rescue. But what should be sequenced, how many reads should be enough, and are there any properties of the sample that might predict the success of the approach?

### Authors' response

We believe there is no formula to compute the minimum number of sequences necessary to determine all the SL couples of any given protein, because it depends of the unknown count of such couples. On the other hand, the Chi^2 ^test imposes a theoretical count in each of the 4 cells of the contingency table greater than 5. And this for each pair of positions studied. So for each actual SL couple, this takes at least 20 sequences complying with the above conditions. But the number of SL couples is of course initially unknown.

### Reviewer's comment

Perhaps some simulations, and repetition of the experiment on the historic data on HIV (what was the year, dataset, or sequencing strategy that for the first time made the synthetic-lethal pairs visible using the method) may provide some guidance?

I declare that I have no competing interests.

### Reviewer's report 2

Reviewer 2: Shamil Sunyaev, Harvard Medical School, Boston, MA, United States

### Reviewer's comment

This is an interesting manuscript on molecular evolution and structural analysis of the HIV protease. The goal of the study is to use co-evolution of amino acid pairs for proposing drug binding sites immune to mutational escape. Although the work is of interest, I have a few comments listed below.

The approach based on the contingency table analysis ignores phylogenetic structure of the dataset. This may lead to false-positive predictions of co-evolved residues. It is clear that the size of the dataset prevents any accurate phylogenetic analysis. However, it should be easier to verify that selected clusters cannot be explained by phylogeny and represent a real signal of co-evolution. At least, the manuscript would benefit from a detailed discussion of this point.

### Authors' response

We read your suggestion with interest. However, it appears impossible to execute the described experiments due to the number of sequences in our dataset (24151 sequences), and the idea of limiting the phylogenetic study to selected clusters is interesting. Still, from an evolutionary point of view, we believe that the positive co-evolution of two sites (both positions always mutate together) is not the same issue as exclusive co-evolution (both positions can mutate but never together) Indeed, when examining the propagation of 2 mutated positions in a tree, two situations can be distinguished: either they are co-present in the majority of the sub-trees, which tends to indicate noise (since there is no segregation); or they are co-present only in the leaves of one part of the tree, which tends to indicate true co-evolution. Inversely, when studying a case of exclusive co-evolution, only one of these two situations is possible. Indeed, by definition of synthetic lethals, both mutations cannot be part of the same sub-tree since their co-presence is lethal. So the tree resulting from this situation is impossible to analyze, since one should analyze all the sub-trees in order to find the missing branches - which corresponds to the statistical study we conducted here.

We have included this paragraph in the discussion sections.

### Reviewer's comment

It is unclear why does the method treat variation in all positions as bi-allelic (mutated or non-mutated genotype). It is possible that taking into account actual amino acid types would be more informative. Again, this point warrants at least a detailed discussion.

### Authors' response

Yes, we totally agree with you and we also thought about this issue. Now to address it, we needed to choose a score matrix. Most score matrixes do take into account the physico-chemical specificity of each amino-acid so as to decide whether the mutated one is similar or not to the initial one. But the mutations involved in resistance to anti-protease drugs do not follow the same rules. Indeed, resistance can be due to the mutation of one amino-acid into another one of very close physico-chemical characteristics. Therefore we do not think using a score matrix of the blosum or Pam type would be more informative.

We have included this paragraph in the discussion sections.

### Reviewer's comment

The manuscript would benefit from the clarification of the main hypothesis. Why would a single mutation at the SL cluster be always insufficient for an efficient escape?

### Authors' response

We do not state that a single mutation at the SL cluster is always insufficient for an efficient escape, then 2 mutations should be necessary for escape. But we state that if several mutations appear in a cluster, this combination is lethal. So, and this is our main hypothesis: increasing the number of drug-docking points, while at the same time decreasing their freedom to co-mutate, does diminish escape possibilities.

### Reviewer's comment

The authors observe co-evolution rather than synthetic lethality. This result can be explained by a very moderate fitness loss rather than lethality. This is a serious complication for the anti-escape strategy. It would be great to discuss this issue.

### Authors' response

To determine whether a situation is of « very moderate fitness » or "lethality", one must examine the p-value associated to the chi^2 ^test: the lower the p-value, the lower the fitness, the closer the situation is to lethality. So to clarify this point, we added all the couples' p-values on figure 2. Additionally, it is important to note that the p-value of a locked set is always lower than or equal to the p-values of its SL couples: p(A inter B) = p(A) + p(B) - p(A union B). So the larger the cluster, the lower the fitness.

### Reviewer's comment

It is of interest that the majority of SL pairs are distant. Possibly, this warrants an additional discussion as well.

### Authors' response

Indeed, out of a total 290 SL couples, 58 are close in space. The co-evolution of positions generally demonstrates a structural or functional link between these positions. Inasmuch as these links appear obvious when two sites are close in space, when the two sites are distant one has to infer long-range interactions.

### Reviewer's report 3

Reviewer 3: Claus Wilke, University of Texas, Center for Computational Biology and Institute for Cell and Molecular Biology, Austin, Texas, United States.

### Reviewer's commen

The authors suggest a novel approach to developing drugs that HIV could not easily evolve resistance to: drugs that target multiple sites, all of which cannot jointly mutate. I think this is a valid concept in principle. The authors then proceed to identify sets of mutations that might be suitable drug targets, through an analysis of covariation in a large alignment of HIV sequences.

By and large, I find the paper a bit thin. The proposed sets of mutations are only candidates, there is no experimental verification showing that they truly cannot mutate together. There is also no demonstration that a drug could actually be made to act at the specific combination of sites proposed. On the upside, the authors are very forthcoming with the weaknesses of the study. They do not oversell their results.

### Authors' response

Are the locked targets we described actually druggable pockets? We studied this using q-site, a software program from the University of Leeds. Q-site takes a 3D structure as input, and outputs its top 10 ligand-binding sites. Out of the top 10 ligand-binding sites thus spotted in the protease, 2 overlap our 3 locked targets (SGI-flap and SGI-fulc). Five out of seven positions in SGI-flap (39, 40, 41, 42, 60) are in the pocket of one of the 10 sites defined by q-site (39, 40, 41, 42, 43, 58, 59, 60, 61, 62). Three out of five positions in SGI-fulc (12, 14, 19) are in the pocket of one of the 10 sites defined by q-site (12, 13, 14, 19, 65, 66, 67, 68).

We have included these results in the manuscript as a new paragraph in the result and in the discussion sections.

### Reviewer's comment

I have one major concern with the definition of synthetic lethals, though. The authors identify them on the basis of a test of association. The null hypothesis in this test is that the frequency with which both sites are mutated is the product of the frequencies with which individual sites are mutated. Any deviation from this null hypothesis, however small, can produce a significant result if the number of observations is sufficiently large. Thus, if site B is only slightly less likely to be mutated when site A has been mutated, the authors' procedure would predict that A and B are a synthetic lethal pair, even though the fitness cost of mutating both A and B may be minute.

### Authors' response

The claim is that chi^2 ^testing on large samples detects the smallest dependency. But since we check the False Discovery Rate and produce a table of p-values, we can determine the validities of the chi^2 ^tests. These p-values appear on Figure 2.

### Reviewer's comment

It seems to me that a better way to define synthetic lethals is to search for pairs of sites that never mutate together, i.e., where the frequency of (1 1) pairs is below the threshold for sequencing errors of (.3%)^2. I suspect, though, that such pairs are exceedingly rare.

A second concern that I have with the method in the present paper is that it does not consider the effect of evolutionary history. It is well known that phylogenetic relationships can produce apparent non-independence of sites. Methods to control for this effect exist and could be used in place of the authors' method, see e.g. Noivirt O, Eisenstein M, Horovitz A. (2005) Detection and reduction of evolutionary noise in correlated mutation analysis. Protein Eng. Des. Sel. 18, 247-253.

### Authors' response

We read the above article with interest. Unfortunately, it seems impossible to execute the experiments described, the number of sequences in our dataset being too large (24,151 sequences). Furthermore, from an evolutionary point of view, we believe that the positive co-evolution of two sites (both positions always mutate together) is not the same issue as exclusive co-evolution (both positions can mutate but never together) Indeed, when examining the propagation of 2 mutated positions in a tree, two situations can be distinguished: either they are co-present in the majority of the sub-trees, which tends to indicate noise (since there is no segregation); or they are co-present only in the leaves of one part of the tree, which tends to indicate true co-evolution. Inversely, when studying a case of exclusive co-evolution, only one of these two situations is possible. Indeed, by definition of synthetic lethals, both mutations cannot be part of the same sub-tree since their co-presence is lethal. So the tree resulting from this situation is impossible to analyze, since one should analyze all the sub-trees in order to find the missing branches - which corresponds to the statistical study we conducted here.

We have included this paragraph in the discussion sections.

### Re-reviewer's report 3

Reviewer 3: Claus Wilke, University of Texas, Center for Computational Biology and Institute for Cell and Molecular Biology, Austin, Texas, United States.

### Reviewer's comment

The authors use the concept of co-lethality of mutations to identify possible drug targets in HIV. I have two major comments.

1. I am concerned about the definition of synthetic lethals. The authors identify them on the basis of a test of association. The null hypothesis in this test is that the frequency with which both sites are mutated is the product of the frequencies with which individual sites are mutated. Any deviation from this null hypothesis, however small, can produce a significant result if the number of observations is sufficiently large. Thus, if site B is only slightly less likely to be mutated when site A has been mutated, the authors' procedure would predict that A and B are a synthetic lethal pair, even though the fitness cost of mutating both A and B may be minute.

2. The method in the present paper does not control for the effect of evolutionary history. It is well known that phylogenetic relationships can produce apparent non-independence of sites. Methods to control for this effect exist and could be used in place of the authors' method, see e.g. Noivirt O, Eisenstein M, Horovitz A. (2005) Detection and reduction of evolutionary noise in correlated mutation analysis. Protein Eng. Des. Sel. 18, 247-253. The authors argue that their data set is too large to control for phylogeny. That may be the case, but it doesn't alter the fact that the data set is likely confounded by phylogeny.
